# Supplementary material for: Natural self-attenuation of pathogenic viruses by deleting the silencing suppressor coding sequence for long-term plant-virus coexistence
Source: PLoS Pathog. 2025 Jun 26;21(6):e1013012. doi: 10.1371/journal.ppat.1013012 (PMC12225820; doi:10.1371/journal.ppat.1013012)
Supplement: S1 Table — (PDF) [file ppat.1013012.s002.pdf]

S1 Table. Primers used in this study

Primer Name	Sequence (5’→3’)	Use
P1′	ATGGCTGCATCATCTAAAGCTGC	RT-PCR: target HCPro1-HCPro2 in ANSSV
P2′	TTTCACCTTTAATTGCTCGCTGG	
P3′	ATGGATGTTGGAGAAGAAAGAAGATTAATGC	RT-PCR: target CP in ANSSV
P4′	AACTGACTTAGCTCCTTCTAGAAACGG	
P5′*	GTATCGTGCTTGATTCTGG	RT-PCR: target *AcActin* transcripts
P6′*	GCTACTCTTGGCTGTCTCC	
P7′	TGGCGTGAGCACTACAAACC	RT-PCR: target HCPro1-HCPro2 in ANRSV
P8′	TTCCTTTCACTCTCCTCCCTGCTAACTTC	
P9′	ATTCCAACAGGGCCAAATAC	RT-PCR: target CP in ANRSV
P10′	GGACCATGTTCATACTCACTAACAT	
SS1-F	AAATTAGAACAAAACAAAACAAAACAAAACAAAC	Genome Cloning: ANSSV-BT23
SS1-R	GCGTGTTCTTTAGCAATTTTCTTCATTAG	
SS2-F	GCGGCTATTTTTGAGAATGATGC	
SS2-R	AGCAAGTTGTAGTAGGCCACTG	
SS3-F	TACAAGGAGGAAACGCAGCTGA	
SS3-R	CACGTCCATTGTCACACCTG	
SS4-F	GGATCCATTTAATTATGGCAAAACCGTGTTA	
SS4-R	TGATGGATTATGTGGTGTCA	
SS5-F	CAAATGATGACATGGTGG	
SS5-R	GTCTTCAAGAACTTGCTCAGT	
SS6-F	GCTGCTTATTGGAAGGATCT	
SS6-R	GGATCTCTAGTTAATTCCTC	
SS7-F	TGTGTGCAATGGCGATGATA	
SS7-R	TTTTTTTTTTTTTTTTTTTTTACCTAAGGGTACTAGGTTCAGTGAG	
SS-5RACE-1R	CAGCATTCCACTTCCATGCAGC	
SS-5RACE-2R	CATGAACTCCTCGCCCCATAAGG	
SS-5RACE-3R	the same sequence with the primer P2′	
SS-3RACE-1F	GATCTGGGGTGAAGAGAGCTATG	
SS-3RACE-2F	GGTATGAACATGGCCCTTCAGGC	
P7′	TGGCGTGAGCACTACAAACC	Genome Cloning: ANRSV-HK23
RS-3550R#	GGCACCAAGAACAAAATTCCAATC	
RS-3460F#	GGAATATGGAGCAGAGTATGGAA	
RS-4750R#	TCCTCAAGCCTTGGCCATGAGTTG	
RS-4650F#	AAGGGAGGGTTCACAGGAGTGTG	
RS-6300R#	TGGCTGTTGAAATTGTGTGTGACCA	
RS-6210F#	CAAGTTGAAGTTCCAGGGTAGCTT	
RS-7520R#	TGGAGCTGCTGTGAAAACTCTGG	
RS-7440F#	GAAGGAAGGAATGCTGGAATTTGGA	
RS-9300R#	GGACCATGTTCATACTCACTAACAT	
RS-5RACE-1R	CGAAGGGCCCAACATCGATCTG	
RS-5RACE-2R	the same sequence with the primer P8′	
RS-5RACE-3R	GACACTCTCTTCCGTCCCAACATAG	
RS-3RACE-1F	TGACCCAAGCAAAGGCAGGAGC	
RS-3RACE-2F	CAGATGGCAATGTATCACAGGGAAC	
5UTR-DP-F1	C/TAAAACAAAA/TCAAAACAAACAAGAAAAGA	Field Survey: flanking HCPro1-HCPro2
P3-DP-R1	GATTCC/TAT/AC/AAGATCC/TTGT/CTCATAATGGCT	
HCPro-DP-F1	G/AAAA/GGATT/CTC/TTTT/CTATGGAGTA	Field Survey: targeting full-length viruses
CP-DP-F2	CAAGT/CATGGAACTGCCATTGAGGCC	Field Survey: targeting viral CP
CP-DP-R1	GTG/TGTG/ATGTCTC/TTCATAACTTGTT/CCC	
SS23-qPCR-F	TGGAGAAGCAGCATCATGGAAT	RT-qPCR: ANSSV-BT23, ANSSV-BT23-HCPro1-2^{17}
SS23-qPCR-R	AAAGTGCATCTTGTGGTAGGGT	
RS23-qPCR-F	ACACTCACGAGGTGGGCAATTA	RT-qPCR: ANSSV-HK23, ANRSV-HK23-HCPro1-2^{ZYZ}
RS23-qPCR-R	TCCTGCCTTTGCTTGGGTCATA	
ANSSV-All-F	AGGAAGTTCATTTCATTTGGAGAGGAAATTAGAACAAAACAAAA CAAAACAAAACAAAC	Development of Full-length cDNA Clone: pSS-BT23
Intron-SOE-2	ccacacatactctttaagtgcatacttacCTTGACTTCCATTCCATT	

Intron-SOE-3	caatctttgaaattgtgcagGAGAGGTTGGATCAAACTTA	
SS-5900R	TGATGGATTATGTGGTGTCA	
SS-4200F	CGCTCTGGGCAGATGACTGTC	
SOE-G-2	GAAAAGTTCTTCTCCTTTACTCATGTCCATTTGGAATTCCTTGTTTG	
SOE-G-5	AACTATACAAAGCCAATAAAGAGTTTCAGATGGATGTTGGAGAAGAAAGAAG	
ANSSV-All-R	TTGAACGATCGTCGACTTTTTTTTTTTTTTTTTTTTACCTAAGGGTACTAGGTTCAGTG	
Intron-SOE-1	AATGGAATGGAAGTCAAGgtaagtatgcacttaaagagtatgtgtgg	
Intron-SOE-4	TAAGTTTGATCCAACCTCTCctgcacaatttcaaagattg	
SOE-G-3	TGCAAACAAGGAATTCCAAATGGACATGAGTAAAGGAGAAGAACTTTTCAC	
SOE-G-4	CCAACATCCATCTGAAACTCTTTATTGGCTTTGTATAGTTCATCCATGC	
Nos-F	GAACCTAGTACCCTTAGGTAAAAAAAAAAAAAAAAAAAAAGTCGACGATCGTTCAAACAT	
e35S-R	TTTCCTCTCCAAATGAAATGAAC	
ANRSV23-All-F	GAAGTTCATTTCATTTGGAGAGGAAATTAAAACAAATTAAAACAAATCAAAACAAACA	Development of Full-length cDNA Clone: pRS-HK23
RSV-5466R	CCAGCTATGATGTATAGTGAAC	
RSV-3689F	CGTGAAAGAATTCGAGGCAGC	
RS23-SOE-G-1R	GTTCTTCTCCTTTACTCATGTCCATTTGAAATTCCTTGCTTGC	
RS23-SOE-G-2F	CAAGGAATTTCAAATGGACATGAGTAAAGGAGAAGAACTT	
ANRSV23-All-R	GAAGTTCATTTCATTTGGAGAGGAAATTAAAACAAATTAAAACAAATCAAAACAAACA	
RS23-SOE-G-1F	CAAGGAATTTCAAATGGACATGAGTAAAGGAGAAGAACTT	
RS23-SOE-G-4[#]	CCAATATCCATCTGGAACTCTTTGCTCGCTTTGTATAGTTCATCCATGC	
RS23-Nos-F	GAACCTATTACCCTTAGGTAAAAAAAAAAAAAAAAAAAAAGTCGACGATCGTTCAAACAT	
SS23-SOE-HP1-F	TGGGGCGAGGAGTTCATGTTCATGACGAGCTGTGGAAAATGGTTGAG	Development of Hybrid Virus Clone: pSS-BT23-HCPro1-2[17]
SS23-SOE-HP2-P3-R	CATCCCAATATGATCGATTTCGTCACTCAATGTTCCTCCAACTCTA	
pCB301-F	TACCCGCCAATATATCCTGTC	
SS23-SOE-HP1-R	CTCAACCATTTTCCACAGCTCGTCATGAACATGAACTCCTCGCCCCAT	
SS23-SOE-HP2-P3-F	TAGAGTTGGAGGAACATTGAGTGACGAAATCGATCATATTGGGATG	
SS23-1300R	GAGCAGCAACCCCATATATAAG	
RS23-SOE-5UTR-HP1-F	GAACCGACTGCAAGAGCAACAATGGCTACACCATCGAAGGCAGC	Development of Hybrid Virus Clone: pRS-HK23-HCPro1-2[ZYZ]
RS23-SOE-HP2-P3-R	GTTAATCTCAGAGTTGAGTGATGCTCCAACTTTAAATTGAAACCAAC	
RS23-SOE-5UTR-HP1-R	GCTGCCTTCGATGGTGTAGCCATTGTTGCTCTTGCAGTCGGTTC	
RS23-SOE-HP2-P3-F	GTTGGTTTCAATTTAAAGTTGGAGCATCACTCAACTCTGAGATTAAC	

Note: The primers indicated with * and # were designed with reference to previous publication (Zhou et al., 2022, New Phytologist; Wang et al., 2021, Frontiers in Microbiology).

References

Zhou, G., Yin, H., Chen, F., Wang, Y., Gao, Q., Yang, F., ... & Wan, Y. (2022). The genome of Areca catechu provides insights into sex determination of monoecious plants. New Phytologist, 236(6), 2327-2343.

Wang, Y., Shen, W., Dai, Z., Gou, B., Liu, H., Hu, W., ... & Cui, H. (2021). Biological and molecular characterization of two closely related arepaviruses and their antagonistic interaction in Nicotiana benthamiana. Frontiers in Microbiology, 12, 755156.
