# Supplementary figures and images for: Natural self-attenuation of pathogenic viruses by deleting the silencing suppressor coding sequence for long-term plant-virus coexistence
Source: PLoS Pathog. 2025 Jun 26;21(6):e1013012. doi: 10.1371/journal.ppat.1013012 (PMC12225820; doi:10.1371/journal.ppat.1013012)

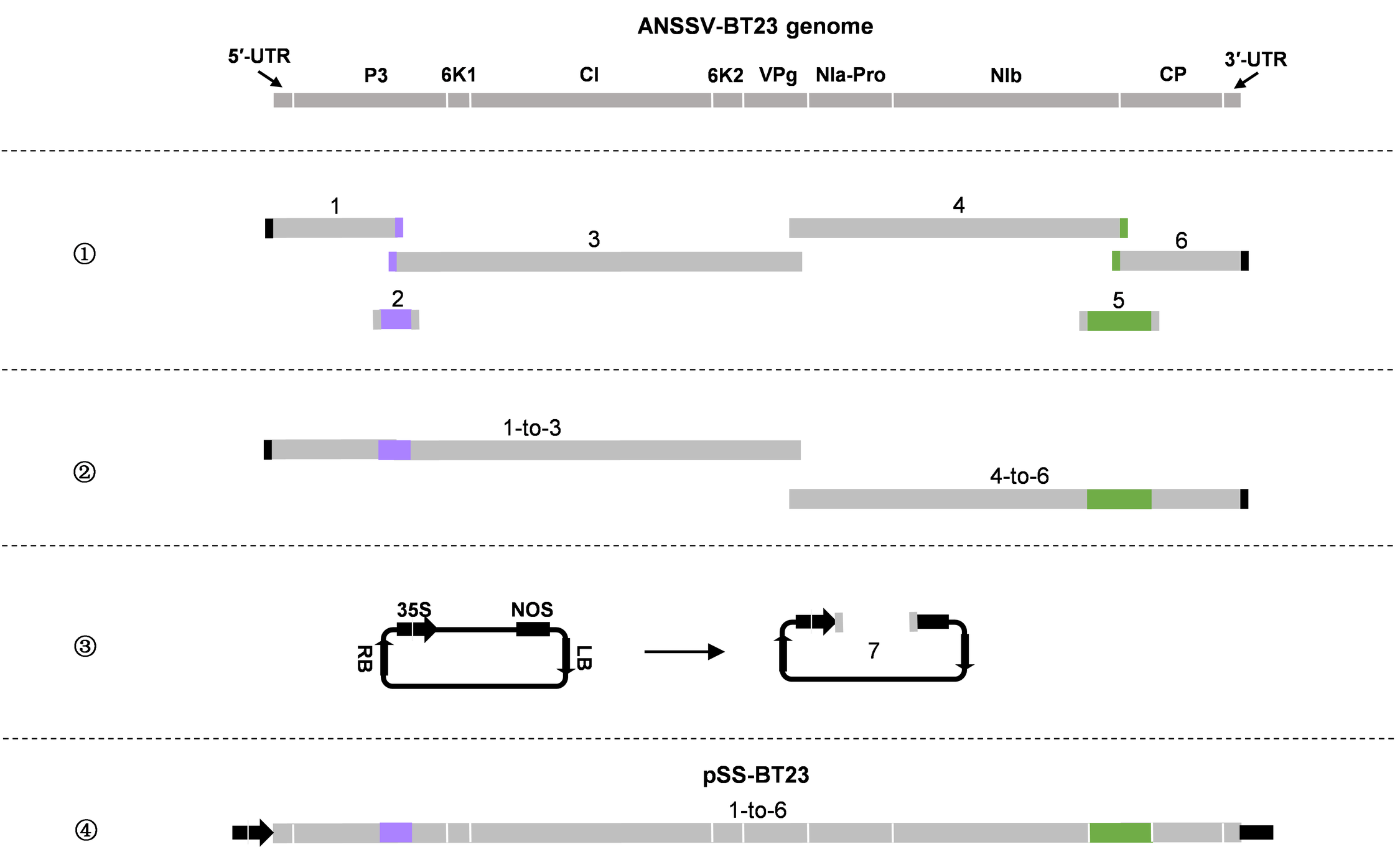

Supplement: S1 Fig — Purple and blue rectangles represent a 220-bp intron 2 of NiR gene (Phaseolus vulgaris) and the complete GFP-coding sequence, respectively. Vertical white lines inside and outside P3-to-CP in the ANSSV-BT23 genome represent NIa-Pro cleavage sites and initiation/ stop codons, respectively. (TIF) [file ppat.1013012.s003.tif]

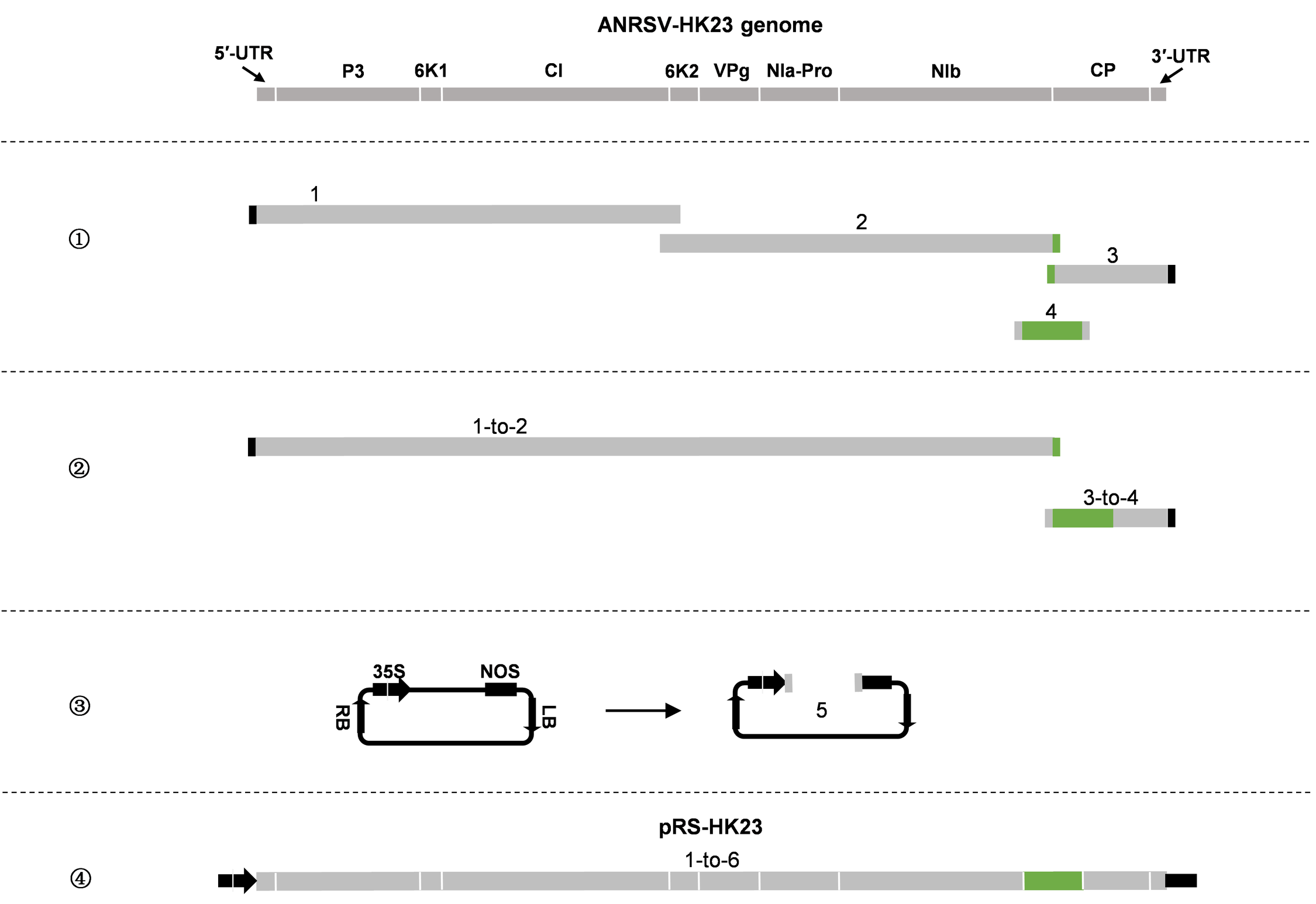

Supplement: S2 Fig — Blue rectangles represent the complete GFP-coding sequence. Vertical white lines inside and outside P3-to-CP represent NIa-Pro cleavage sites, and initiation/ stop codons, respectively. (TIF) [file ppat.1013012.s004.tif]

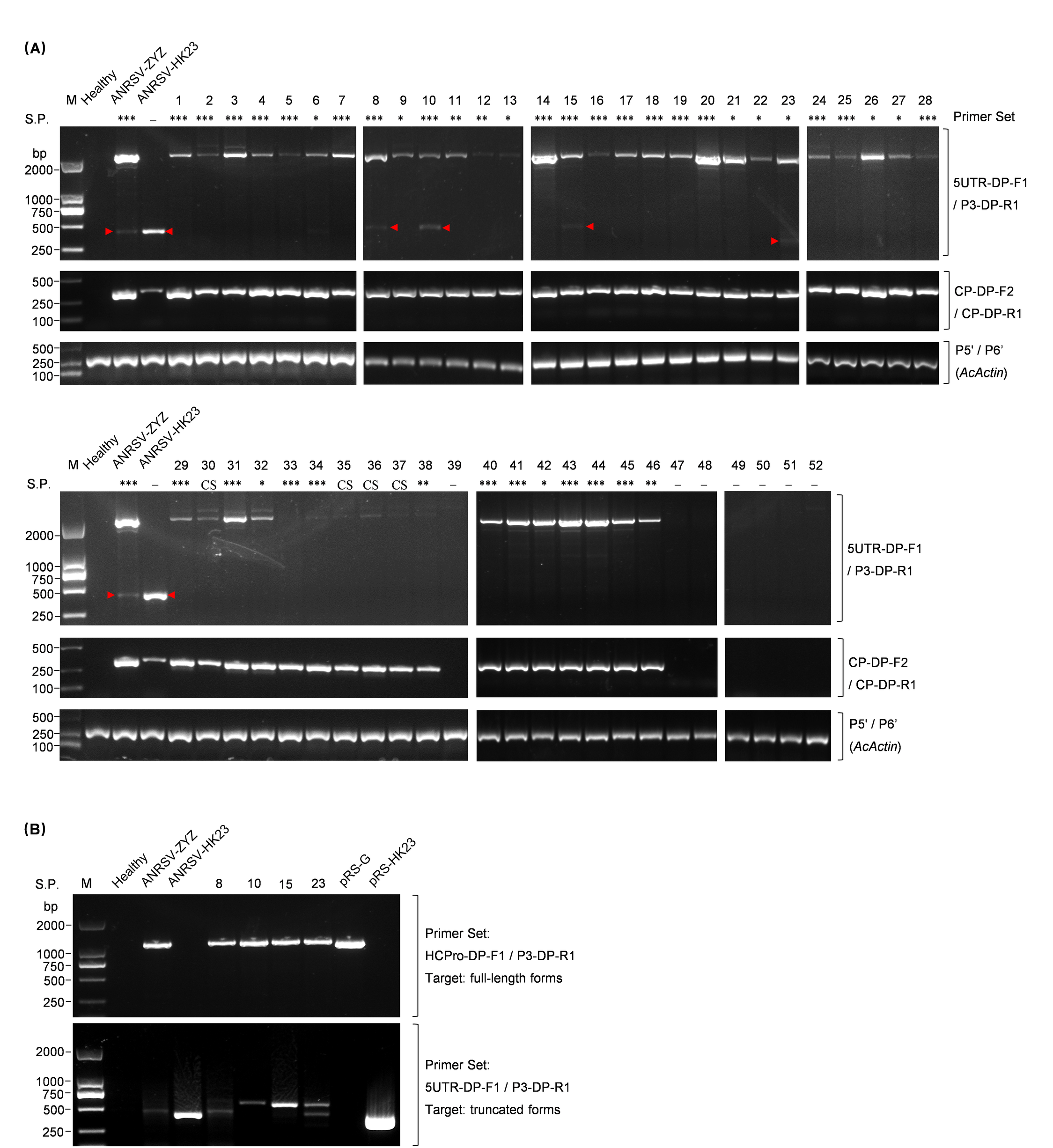

Supplement: S3 Fig — (A) RT-PCR analysis demonstrates the co-existence of full-length and leader protease-less isolates in the same trees. Adult trees in the field were classified into five groups based on disease symptomatology: -, symptomless; CS, chlorotic spots in top leaves; the symbols *, ** and *** indicate that approximately one third, half, and two third of total leaves in the trees display severe necrotic spots, respectively. A newly-expanded leaf from each tree was sampled for total RNA extraction, and RT-PCR with the indicated primer sets was carried out. The presence of ANSSV/ ANRSV was assessed with a pair of degenerate primers: CP-DP-F2/ CP-DP-R1 (middle panels). The region spanning HCPro1-HCPro2 was amplified with another primer of degenerated primers: 5UTR-DP-F1/ P3-DP-R1 (upper panels). The detection of AcActin transcripts in each sample was used as internal control (lower panels). A leaf from an areca palm tree infected with ANRSV-ZYZ and showing severe foliage necrosis was harvested in Dingan, Hainan, in 2020 [47]. This tissue was also used to prepare total RNA for RT-PCR. (B) RT-PCR-based assay to distinguish between full-length viruses and shorter versions within the same trees. The PCRs with primer set HCPro-DP-F1/P3-DP-R1 (2 × Taq PCR MasterMix II; 30 cycles, 1.5-min extension/ cycle) produce a long amplicon, indicating the presence of full-length viruses. The primer set 5UTR-DP-F1/ P3-DP-R1 were used to detect the shorter versions with a short extension time (2 × Taq PCR MasterMix II; 28 cycles, 30-sec extension/ cycle). Two plasmids, pRS-G [47] and pRS-HK23 in this study, harboring the cDNAs corresponding to the full-length and the shorter isolates of ANRSV, respectively, were included as the internal controls. (TIF) [file ppat.1013012.s005.tif]

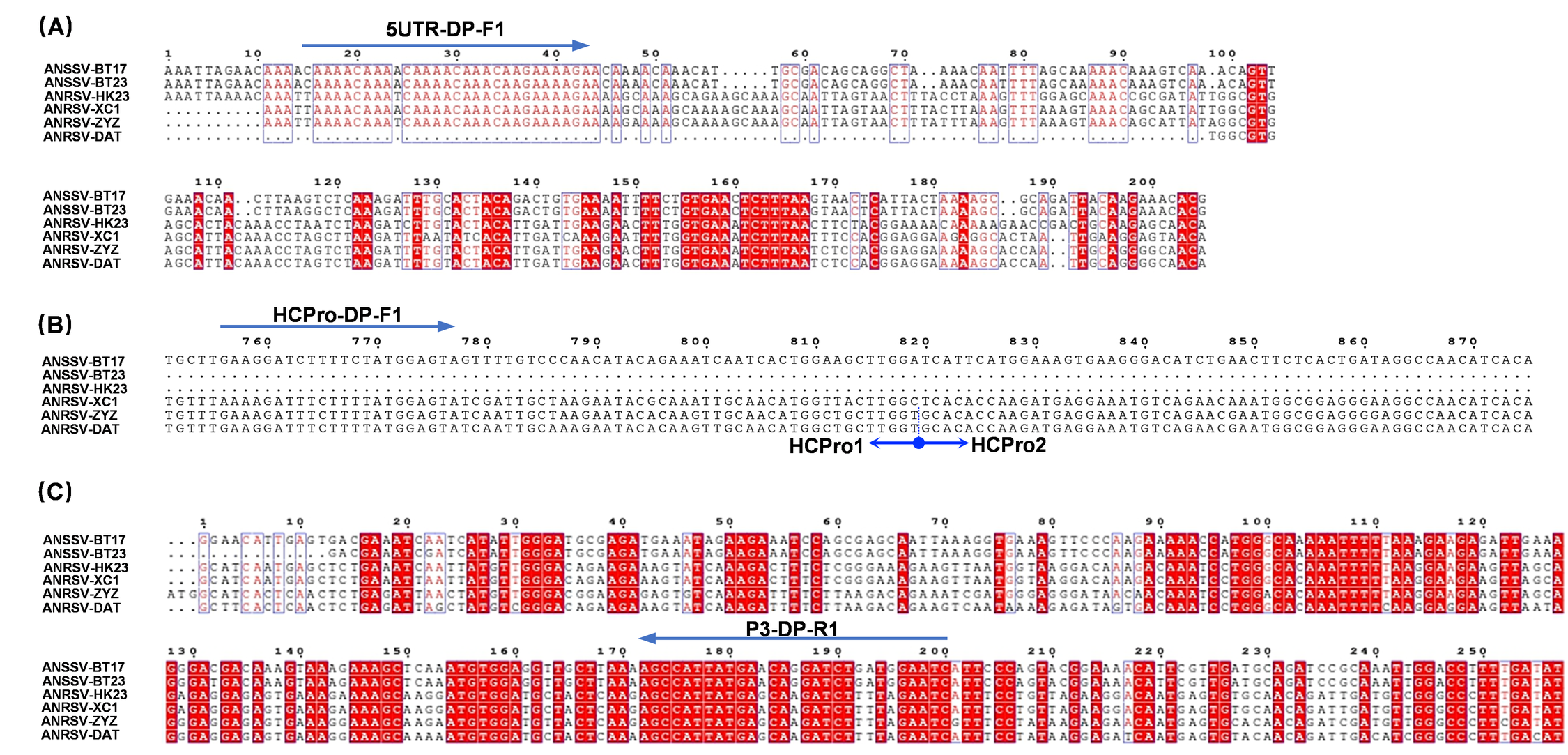

Supplement: S4 Fig — Multiple alignment of 5’ UTR (A), HCPro1-HCPro2 (B), or P3 (C) sequences across ANSSV and ANRSV isolates. The aligned regions corresponding to degenerate primers 5UTR-DP-F1 (A), HCPro-DP-F1 (B), and P3-DP-R1 (C), are shown. Except for the sequences of ANSSV-BT23 (PQ867792) and ANRSV-HK23 (PQ867793) obtained in this study, the remaining ones were retrieved from the GenBank database: ANSSV-HNBT (namely ANSSV-BT17 in this study) (MH330686), ANRSV-XC1 (MH395371), and ANRSV-ZYZ (MZ209276). Identical residues are shown with white letters in red background, whereas conserved substitutions are displayed with red letters in blue boxes. (TIF) [file ppat.1013012.s006.tif]

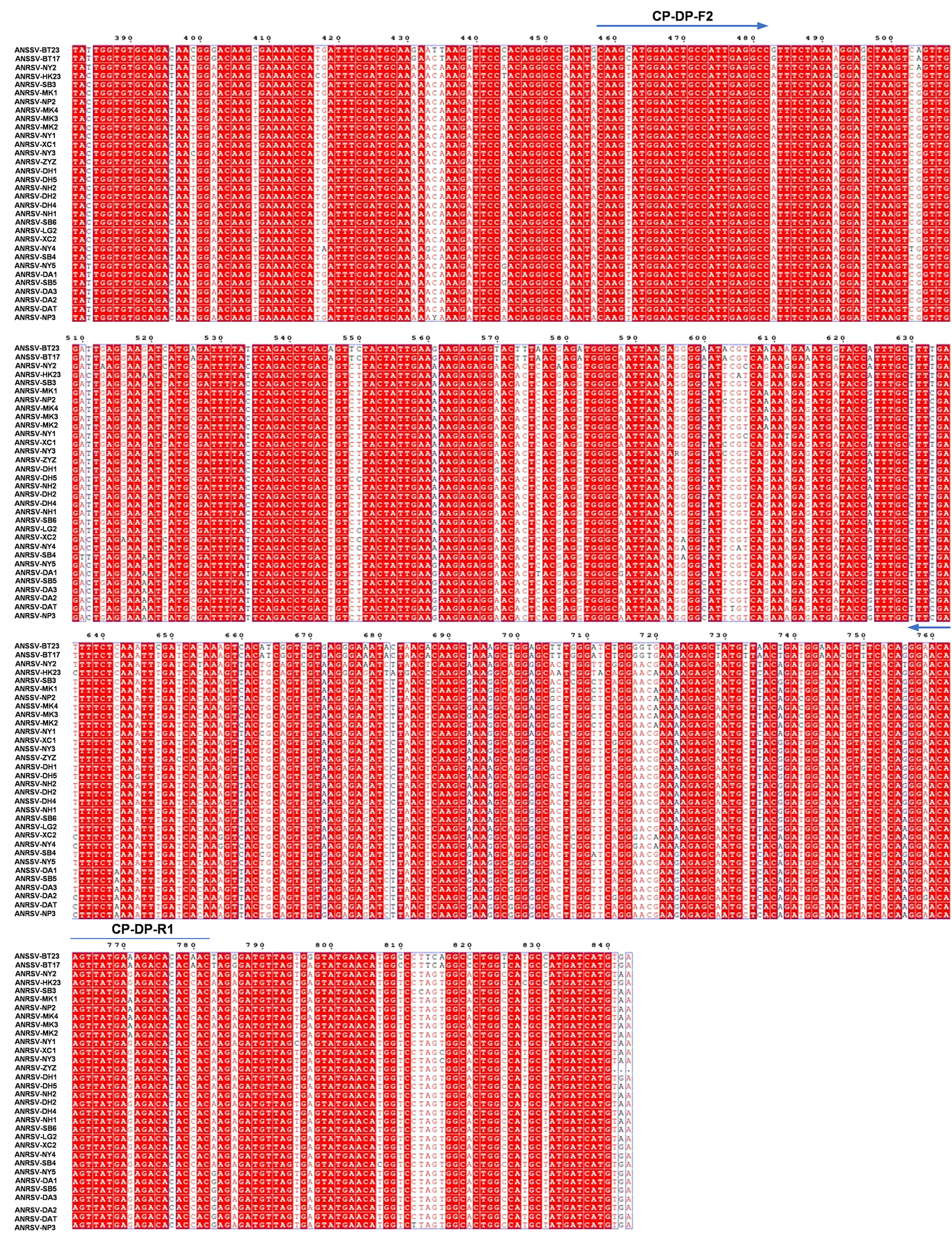

Supplement: S5 Fig — Multiple alignment of CP sequences from ANSSV and ANRSV isolates, highlighting the regions corresponding to the pair of degenerate primers CP-DP-F2 and CP-DP-R1. Except for the sequences of ANSSV-BT23 and ANRSV-HK23 obtained in this study, the others were retrieved from the GenBank database: ANSSV-BT17 (MH330686), ANRSV-XC1 (MH395371), ANRSV-NY2 (MH395387), ANRSV-MK1 (MH395380), ANRSV-NP2 (MH425893), ANRSV-MK4 (MH395383), ANRSV-MK3 (MH395382), ANRSV-MK2 (MH395381), ANRSV-NY1 (MH395386), ANRSV-NY3 (MH425891), ANRSV-ZYZ (MZ209276), ANRSV-DH1 (MH395375), ANRSV-DH5 (MH395378), ANRSV-DH2 (MH395376), ANRSV-DH4 (MH395377), ANRSV-NH2 (MH395385), ANRSV-NH1 (MH395384), ANRSV-SB6 (MH395393), ANRSV-LG2 (MH395379), ANRSV-XC2 (MH425890), ANRSV-NY4 (MH395388), ANRSV-NY5 (MH395389), ANRSV-SB4 (MH395391), ANRSV-SB5 (MH395392), ANRSV-DA1 (MH395372), ANRSV-DA2 (MH395373), ANRSV-DA3 (MH395374), ANRSV-DAT (MW282956), ANRSV-NP3 (MH425894), ANRSV-SB3 (MH395390). Identical nucleotides are shown with white letters in red background, whereas conserved substitutions are displayed with red letters in blue boxes. (TIF) [file ppat.1013012.s007.tif]

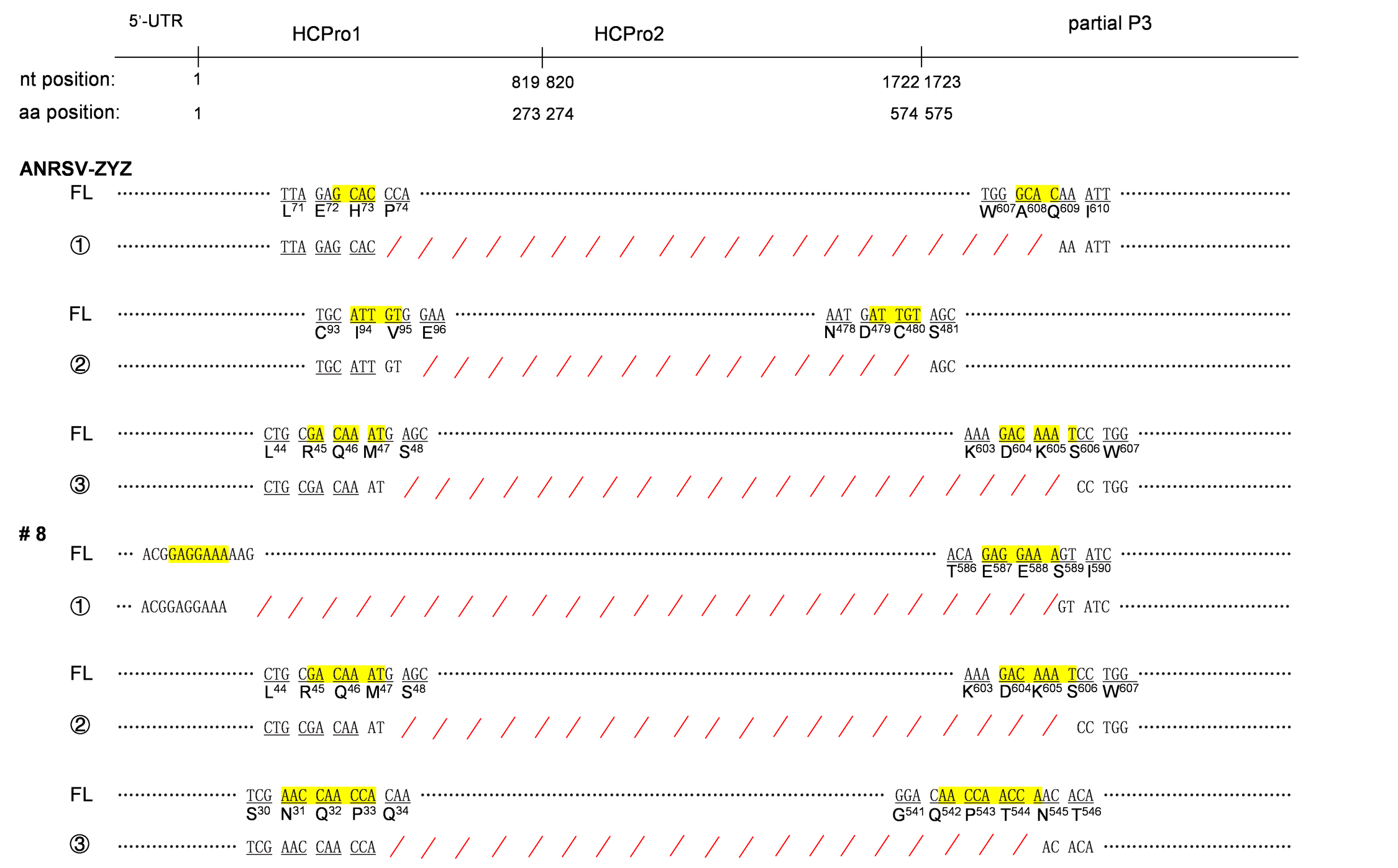

Supplement: S6 Fig — For tree #8 and the one infected with ANRSV-ZYZ, both the long and short amplicons (S3 Fig) were cloned. Plasmids from three independent colonies per plate were sequenced. Sequence from clones corresponding to the long amplicons were identical across samples. Nucleotide sequences of the long (FL) and various short (rounded numbers) amplicons were aligned. Short repeated sequences flanking the deleted fragments in the long amplicon are highlighted in yellow, while deleted regions in the short amplicons are marked with red slant lines. (TIF) [file ppat.1013012.s008.tif]
